# Clinical chemistry profiles in injection heroin users from Coastal Region, Kenya

**DOI:** 10.1186/1472-6890-14-32

**Published:** 2014-07-09

**Authors:** Tom Were, Jesca O Wesongah, Elly Munde, Collins Ouma, Titus M Kahiga, Francisca Ongecha-Owuor, James N Kiarie, Aabid A Ahmed, Ernest P Makokha, Valentine Budambula

**Affiliations:** 1 Department of Clinical Medicine, University of Kabianga, P. O. Box 2030–20200, Kericho, Kenya; 2 Department of Medical Laboratory Sciences, Jomo Kenyatta University of Agriculture and Technology, Juja, Kenya; 3 Department of Biomedical Sciences and Technology, Maseno University, Maseno, Kenya; 4 Department of Pharmacy and Complementary Medicine, Kenyatta University, Nairobi, Kenya; 5 Department of Medicine, Therapeutics, Dermatology and Psychiatry, Kenyatta University, Nairobi, Kenya; 6 Department of Obstetrics and Gynaecology, University of Nairobi, Nairobi, Kenya; 7 Bomu Hospital, Mombasa, Kenya; 8 Centre for Virus Research, Kenya Medical Research Institute, Nairobi, Kenya; 9 Department of Environment and Health Sciences, Technical University of Mombasa, Mombasa, Kenya

**Keywords:** Injection heroin user, Clinical chemistry markers, HIV-1 infection, Anti-retroviral treatment

## Abstract

**Background:**

Although the co-burden of injection drug use and HIV is increasing in Africa, little is known about the laboratory markers of injection drug use and anti-retroviral treatment (ART) in Kenyan injection drug users. This study, therefore, aimed at determining the clinical chemistry profiles and identifying the key laboratory markers of HIV infection during ART in injection heroin users (IHUs).

**Methods:**

Clinical chemistry measurements were performed on serum samples collected from HIV-1 infected ART-experienced (n = 22), naive (n = 16) and HIV-1 negative (n = 23) IHUs, and healthy controls (n = 15) from Mombasa, coastal Kenya.

**Results:**

HIV uninfected IHUs had lower alanine aminotransferase (ALT) levels (*P* = 0.023) as ART-exposed IHUs exhibited lower albumin (*P* = 0.014) and higher AST to platelet index (APRI) (*P* < 0.0001). All IHUs presented with lower aspartate aminotransferase to ALT values (*P* = 0.001) and higher C-reactive protein (CRP) levels (*P* = 0.002). ART-naive IHUs had higher globulin levels (*P* = 0.013) while ART-experienced and naive IHUs had higher albumin to total protein (*P* < 0.0001) and albumin to globulin (*P* < 0.0001) values. In addition, CD4+ T cells correlated with ALT (ρ = −0.522, *P* = 0.011) and CRP (rho, ρ = 0.529, *P* = 0.011) in HIV negative and ART-experienced IHUs, respectively. HIV-1 viral load correlated with albumin to globulin index in ART-experienced (ρ = −0.468, *P* = 0.037) and naive (ρ = −0.554, *P* = 0.040) IHUs; and with albumin to total protein index (ρ = −0.554, *P* = 0.040) and globulin (ρ = 0.570, *P* = 0.033) in ART-naive IHUs.

**Conclusion:**

Absolute ALT, albumin, globulin, and CRP measurements in combination with APRI, AST to ALT, albumin to total protein and albumin to globulin indices may be useful laboratory markers for screening IHUs for initiating and monitoring treatment.

## Background

Of the 14 million people who inject drugs worldwide, 1.64 million are HIV infected [1]. In addition, more than 40% of new HIV infections in the world are attributed to injection drug use [2]. HIV sero-prevalence rates among injection drug users (IDUs) in Kenya are exceedingly high at 18%, and at least, 17% of new HIV infections in the country are linked to injection drug use [3]. Moreover, the rates of HIV transmission in injection drug users in the country are higher relative to the spread in the general population [4], suggesting that injection drugs and substance use are important in promoting the spread of HIV in Kenya.

Although CD4+ T cell measurements are commonly used in initiating and monitoring disease progression and treatment in HIV-1 infected individuals [5], other biomarkers may not be specific to HIV infection due to concomitant illicit drug and poly-substance use among injection drug users. Since, addictive drugs such as opioids largely cause persistent immune stimulation inducing chronic inflammation [6], while anti-retroviral therapy cumulatively cause hepatotoxic injury [7], it is likely that HIV-1 infected injection drug users progressively develop intense inflammation and hepatotoxicity that can be indirectly assessed through determining hepatic functionality.

Injection drugs usually cause chronic liver degeneration and marked derangements in hepatic synthetic functions [6,8,9]. Elevated alanine aminotransferase (ALT), aspartate aminotransferase (AST), globulin, aspartate aminotransferase to platelet index (APRI), and C-reactive protein (CRP) levels are linked to injection drug use in both HIV infected and uninfected individuals [10-14]. In contrast, addictive heroin doses promote reductions in hepatic albumin synthesis [15]. Taken together, these studies suggest increased inflammatory-mediated hepatic derangements in both HIV infected and uninfected injection drug users.

However, no studies to date have investigated clinical chemistry profiles in injection heroin users, resident at coastal Mombasa, Kenya. The identification of clinical chemistry laboratory markers in this sub-population is of great importance, since it can assist in the clinical management of HIV in injection drug users. The current study, therefore, examined clinical chemistry laboratory markers in HIV-1 infected ART-experienced, naïve and HIV-1 non-infected injection heroin users, and healthy controls.

## Methods

### Study site and population

This cross-sectional clinical laboratory study was conducted as part of a larger study investigating the microbiological and immunological determinants of HIV infection among adult injection drug users. The study was undertaken at Bomu Hospital, a social enterprise facility in Mombasa, a coastal region of Kenya. Mombasa has a large injection drug using population of about 26,000, with heroin being the predominant injection drug [3]. Injection drug users were recruited via respondent driven sampling, snowball and makeshift outreach methods. Only individuals (age ≥18 yrs.) exhibiting needle scars, reporting injection heroin use at least once in the previous month and providing written informed consent were recruited into the study. Healthy controls were recruited from among HIV negative individuals presenting no evidence of illness and history of drug use.

### Sample collection

From each injection drug user and healthy control, 10 ml of venous blood was collected. Of this, 5 ml was placed into both the EDTA and plain BD vacutainer® tubes (BD, Franklin Lakes, USA). The EDTA blood samples were used immediately after collection for hematological measurements and the enumeration of CD4+ T cells. Serum was obtained from clotted blood after clot retraction and centrifugation at 15,000 r.p.m for 10 minutes. The serum samples were used for the determinations of clinical chemistry analytes, HIV and hepatitis virus sero-testing, and viral loads.

### HIV-1 diagnosis

HIV-1 testing was performed using rapid immunochromatographic tests, Determine™ (Abbott Laboratories, Tokyo, Japan) and Unigold™ (Trinity Biotech Plc, Bray, Ireland). Study participants with positive results for both Determine and Unigold were considered HIV infected based on the Kenyan national HIV testing algorithm [16].

### HBV and HCV testing

Sero-diagnosis for the presence of hepatitis B virus (HBV) and hepatitis C virus (HCV) infections was performed using the one-step HBV-5 panel, and anti-HCV rapid diagnostic tests, respectively (Healthaw Medical limited, Hangzhou, China). Serum samples were tested for sero-reactivity against the HBV panel markers and anti-HCV. HBsAg and anti-HCV reactive individuals were considered sero-positive for HBV and/or HCV infection.

### Clinical chemistry measurements

Direct clinical chemistry determinations were performed for ALT, AST, gamma-glutamyl transpeptidase (GGT), total protein, albumin, creatinine, urea, total cholesterol, high density lipoprotein (HDL), CRP, phosphocreatine kinase isoenzymes (CK-MB), and vitamin D using the automated clinical chemistry analyzer (Roche COBAS® 6000, Lausanne, Switzerland). Globulin was calculated by subtracting albumin concentrations from the total protein levels and the albumin to globulin ratio >1 was considered normal [17]. The reference values were based on our laboratory established reference ranges. APRI was determined as previously described [18], with AST upper limit values from laboratory established reference values (<37 U/L).

### CD4+ T cell enumerations

Baseline CD4+ T cell counts were determined in an automated fashion using the BD FACSCalibur flow cytometer (Becton-Dickinson™, Franklin Lakes, USA). Briefly, 5.0 μl of EDTA blood samples were placed in a tube and RBC lysis buffer added. After 5 minute incubation, the cells were washed and fluorescent-tagged antibodies (anti-CD3, anti-CD4, and anti-CD45) were added. The cells were incubated for 30 minutes after which the samples were washed and the CD4+ T cells enumerated on the flow cytometer.

### HIV-1 viral load

HIV-1 viral loads were determined using the automated Abbott m2000 System according to the manufacturer’s instructions (Abbott Molecular Inc., Illinois, USA). Briefly, RNA was extracted from 0.2 ml serum samples and reverse-transcribed into cDNA. The cDNA was amplified using HIV-1-specific and internal control primers. Fluorescence intensity of the HIV-1 probe was converted into viral loads by the analyzer.

### Ethical considerations

Ethical approval for the study was obtained from Kenyatta University Ethical Review Committee. Each participant gave written informed consent prior to enrolment into the study. Confidentiality of the study participant’s information was ensured throughout the study by coding and limiting accessibility of the study information. The study participants benefited from free health education on HIV, tuberculosis (TB), hepatitis B and C, sexually transmitted infections, hygiene and nutrition. HIV-positive, TB-positive and HIV-TB co-infected individuals were referred to the comprehensive care centers at Bomu Hospital or the Coast General Provincial Hospital for treatment, support and care.

### Statistical analysis

Statistical analysis was conducted using IBM® SPSS Statistics 19.0 (SPSS Inc. Chicago, USA). Continuous data (age, and laboratory measures) summarized as medians (IQR) and categorical data (gender, duration of injection, and non-injection drugs) presented as proportions were tabulated. Differences in the proportions were determined using the chi-square tests. Statistical comparisons of the continuous data across the study groups were performed using non-parametric ANOVA (Kruskal Wallis) tests followed by Bonferroni post-hoc corrections for multiple comparisons. Spearman’s rank correlation tests were used to determine the associations of clinical chemistry measures with CD4+ T cells and viral loads within the study groups. A two-sided probability value <0.05 was considered statistically significant. Bonferroni correction for multiple comparisons were determined by dividing the criterion *P* < 0.05 by the total number of the study groups (*P* < 0.05/4 = *P* < 0.0125). The *P* < 0.0125 was then used for statistical inferences following between-group Mann Whitney U comparisons.

## Results

### Demographic and laboratory characteristics of the study participants

The study population comprised both male (n = 39) and female (n = 37) adult participants. These comprised of HIV-1 infected ART-experienced (n = 22), naive (n = 16) and HIV-1 non-infected (n = 23) injection heroin users, and healthy controls (HC, n = 15). Table 1 summarizes the clinical, laboratory and demographic characteristics of the study participants. The ages of the study participants were significantly different (*P* = 0.008). Additionally, between-group analysis showed that the anti-retroviral treatment-exposed injection heroin users were relatively older than the healthy controls (*P* = 0.003). The proportions of male and female participants were comparable across the study groups (*P* = 0.351). Use of heroin as an injection drug was common in all the injection heroin users (*P* = 0.999). As non-injection drugs; the use of *bhang*, rohypnol, cigarettes, cocktail and alcohol were not significantly different among the injection heroin users (*P* = 0.584, *P* = 0.338, *P* = 0.972, *P* = 0.351 and *P* = 0.076, respectively). Across group analysis revealed a statistically significant difference in the CD4+ T cell counts/μL (*P* < 0.0001) with the anti-retroviral treatment-exposed (median, 301; IQR, 426) and treatment-naive (median, 448; IQR, 337) groups presenting with lower counts in comparison to HIV uninfected injection heroin users (median, 986; IQR, 474) and healthy controls (median, 755; IQR 463) (*P* < 0.0001 for all between-group comparisons). The lower limit of HIV-1 viral load quantification was 150 (2.18 log_10_) copies/ml of serum sample, and median viral loads were comparable between both anti-retroviral treatment-naive and anti-retroviral treatment-exposed individuals (*P* = 0.163) (Table 1).

**Table 1:** Demographic, clinical and laboratory characteristics of the study participants

**Characteristic**	**HIV-1 uninfected**		**HIV-1 infected**		***P***
	**HC, n = 15**	**IHUs n = 23**	**ART-naive IHUs, n = 16**	**ART-exposed IHUs, n = 22**	
Age, yrs.	25.6 (8.7)	29.2 (7.1)	31.2 (5.9)	33.1 (8.5)^a^	**0.008**
Female/male, (%)	40.0/60.0	39.1/60.9	50.0/50.0	63.6/36.4	0.351
Injection drugs, n (%)					
Heroin	-	23 (100.0)	16 (100.0)	22 (100.0)	0.999
Diazepam	-	0 (0.0)	3 (18.8)	2 (9.1)	-
Duration of injection, n (%)					
<1 yr.	-	12 (52.2)	2 (12.5)	4 (18.2)	-
1-3 yrs.	-	7 (30.4)	9 (56.2)	5 (22.7)	
>3 yrs.	-	4 (17.4)	5 (31.3)	13 (59.1)	
Non-injection drugs, n (%)					
Bhang	-	8 (34.8)	7 (43.8)	11 (50.0)	0.584
Brown sugar	-	3 (13.1)	2 (12.5)	5 (22.7)	-
Rohypnol	-	12 (52.2)	9 (56.2)	16 (72.7)	0.338
Cigarettes	-	18 (78.3)	12 (75.0)	17 (77.3)	0.972
Khat	-	5 (21.7)	3 (18.8)	8 (36.4)	-
Cocktail	-	5 (21.7)	6 (37.5)	9 (40.9)	0.351
Alcohol	-	8 (34.8)	9 (56.2)	15 (68.2)	0.076
CD4+ T cell count/μl	755 (463)	986 (474)	448 (337)^a,b^	301 (426)^a,b^	**<0.0001**
Viral loads, copies/μl	-	-	13,470 (81,176)	150 (17,761)	0.163
Hepatitis B	-	1 (4.3)	2 (12.5)	1 (4.5)	-
Hepatitis C	-	1 (4.3)	3 (18.8)	5 (22.7)	-

Data shown are medians (IQR) unless indicated. HC, healthy controls. ART, anti-retroviral treatment. HIV-1, human immunodeficiency virus-1. IHUs, injection heroin users. Brown sugar, crude heroin. Cocktail, cigarette and bhang mixture. Data analysis was conducted using chi-square for proportions; and Kruskal Wallis tests for continuous data. Following the Kruskal Wallis tests, post-hoc Bonferroni corrections for multiple comparisons were performed based on the Mann Whitney between-group tests (significant at *P* < 0.0125). ^a^ART-exposed and ART-naive IHUs vs. HC, *P* < 0.01. ^b^ART-exposed and ART-naive IHUs vs. HIV-1 uninfected IHUs, *P* < 0.0001. Values in bold are statistically significant at *P*-values indicated.

### Clinical chemistry measurements

While across group analysis did not show significant differences in the levels of phosphocreatine kinase isoenzymes (CK-MB) (*P* = 0.964), the levels of CRP differed significantly across the study groups (*P* = 0.002). Subsequent post-hoc corrections revealed significantly higher levels of CRP in anti-retroviral treatment-exposed (median, 4.0 mg/L; IQR, 7.8; *P* = 0.001), anti-retroviral treatment-naive (median, 3.8 mg/L; IQR, 14.2; *P* < 0.0001) and in HIV-1 uninfected injection heroin users (median, 6.1 mg/L; IQR, 8.4; *P* = 0.001) in comparison to healthy controls (median, 0.7 mg/L; IQR, 0.9) signifying the importance of CRP as an inflammation marker of injection heroin use.

Additional analyses revealed statistical differences in the activity of ALT among the study groups (*P* = 0.023). Between-group tests showed lower ALT levels (U/L) in HIV uninfected injection heroin users (median, 11.0; IQR, 5.5) compared to healthy controls (median, 13.7; IQR, 12.7; *P* = 0.005). However, across group analysis did not reveal any differences in the activity of AST and GGT among the study groups (*P* = 0.341 and *P* = 0.504, respectively). Furthermore, no statistical differences were found in the levels of total cholesterol, HDL cholesterol and the ratio of total cholesterol to HDL cholesterol across the study groups (*P* = 0.176, *P* = 0.828 and *P* = 0.117, respectively). The levels of total protein, creatinine, urea and vitamin D were also comparable across the study groups (*P* = 0.147, *P* = 0.840, *P* = 0.135 and *P* = 0.064, respectively). Across group comparison of albumin levels (g/L) further revealed that there were significant differences (*P* = 0.014), with a further between-group analyses indicating that in anti-retroviral treatment-exposed injection heroin users, albumin levels (median, 41.1; IQR, 10.5) were significantly lower compared to the healthy controls (median, 48.0; IQR, 3.7; *P* < 0.0001). Further analysis indicated that the levels of globulin (g/L) in the anti-retroviral treatment-naive injection heroin users (median, 51.2; IQR, 17.5) were higher compared to the healthy controls (median, 35.1; IQR, 11.1; *P* < 0.0001) and the HIV uninfected injection heroin users (median, 36.6; IQR 10.4; *P* < 0.0001) (Table 2).

**Table 2:** Clinical chemistry measurements of the study participants

**Marker**	**HIV-1 uninfected**		**HIV-1 infected**		***P***
	**HC, n = 15**	**IHUs, n = 23**	**ART-naive IHUs, n = 16**	**ART-exposed IHUs, n = 22**	
CK-MB, U/L	18.0 (15.0)	17.0 (12.0)	17.5 (16.3)	18.0 (26.8)	0.946
CRP, mg/L	0.7 (0.9)	6.1 (8.4)^a^	3.8 (14.2)^a^	4.0 (7.8)^a^	**0.002**
CRP > 5.0 mg/L, n (%)	1 (6.7)	14 (60.9)	7 (43.8)	9 (40.9)	**-**
ALT, U/L	13.7 (12.7)	11.0 (5.5)^a^	10.7 (5.4)	15.2 (11.0)	**0.023**
M > 40; F > 31, n (%)	2 (13.3)	0 (0.0)	1 (6.3)	2 (9.1)	-
AST, U/L	19.5 (9.4)	21.7 (13.0)	22.4 (10.8)	24.9 (14.1)	0.341
AST/ALT	1.1 (0.7)	1.8 (1.0)^a^	2.1 (0.9)^a^	1.6 (0.8)^a^	**0.001**
AST/ALT ≥2.0, n (%)	0 (0.0)	10 (43.5)	10 (62.5)	6 (27.3)	**-**
APRI	0.14 (0.10)	0.23 (0.20)	0.19 (0.10)	0.25 (0.20)^a^	**0.005**
APRI ≥0.5, n (%)	0 (0.0)	0 (0.0)	1 (6.3)	4 (18.2)	-
GGT, U/L	23.0 (28.1)	27.0 (20.1)	27.5 (39.0)	41.5 (45.0)	0.504
TC, mmol/L	5.0 (1.5)	4.1 (2.0)	4.3 (1.9)	4.2 (1.6)	0.176
HDL, mmol/L	1.3 (0.5)	1.3 (0.7)	1.1 (0.7)	1.1 (0.8)	0.828
TC/HDL	3.5 (1.0)	3.3 (1.7)	3.9 (1.0)	4.0 (1.5)	0.117
ALB, g/L	48.0 (3.7)	45.3 (17.8)	41.5 (14.3)	41.1 (10.5)^a^	**0.014**
ALB <32.0 g/L, n (%)	0 (0.0)	1 (4.3)	2 (12.5)	2 (9.1)	-
Total PROT, g/L	82.3 (15.9)	84.4 (22.8)	90.2 (27.7)	82.0 (27.8)	0.147
ALB/PROT	0.6 (0.1)	0.6 (0.1)	0.4 (0.2)^a,b^	0.5 (0.1)^a,b^	**<0.0001**
GLB, g/L	35.1 (11.1)	36.6 (10.4)	51.2 (17.5)^a,b^	41.5 (32.9)	**0.013**
ALB/GLB	1.4 (0.3)	1.5 (0.4)	0.8 (0.6)^a,b^	1.0 (0.5)^a,b^	**<0.0001**
ALB/GLB ≤1, n (%)	0 (0.0)	3 (13.0)	10 (62.5)	11 (50.0)	-
Creatinine, μmol/L	80.0 (23.0)	72.0 (30.0)	75.0 (30.0)	81.0 (29.0)	0.840
Urea, mmol/L	3.5 (1.2)	2.9 (2.5)	2.8 (0.6)	3.0 (1.4)	0.135
Vitamin D, ng/ml	30.7 (8.2)	32.0 (15.0)	35.3 (23.3)	38.3 (13.3)	0.064

Data presented are medians (IQR) unless indicated. HC, healthy controls. ART, anti-retroviral treatment. HIV-1, human immunodeficiency virus-1. IHUs, injection heroin users. CK-MB, phosphocreatine kinase isoenzymes. CRP, C-reactive protein. AST, aspartate aminotransferase. ALT, alanine aminotransferase. APRI, aspartate to platelet index. GGT, gamma-glutamyl transpeptidase. TC, total cholesterol. HDL, high density lipoprotein cholesterol. PROT, protein. ALB, albumin. GLB, globulin. Vitamin D3, 1,25-dihydroxyvitamin D. Data analysis was conducted using chi-square for proportions; and Kruskal Wallis tests for continuous data. Following the Kruskal Wallis tests, post-hoc Bonferroni corrections for multiple comparisons were performed based on the Mann Whitney between-group tests (significant at *P* < 0.0125). ^a^ART-exposed, ART-naive or HIV uninfected IHUs vs. HC, *P* < 0.01. ^b^ART-exposed and ART-naive IHUs vs. HIV-1 uninfected IHUs, *P* < 0.01. Values in bold are statistically significant at *P*-values indicated.

### AST, ALT, albumin, globulin and platelet ratios

Values for the AST to ALT ratio varied across the groups (*P* = 0.001) and were higher in the anti-retroviral treatment-exposed injection heroin users (median, 1.6; IQR, 0.8; *P* < 0.01), anti-retroviral treatment-naive injection heroin users (median, 2.1; IQR, 0.9; *P* < 0.01) and HIV negative injection heroin users (median, 1.8; IQR, 1.0; *P* < 0.01) relative to healthy controls (median, 1.1; IQR, 0.7). In addition, at least a quarter of the anti-retroviral treatment-exposed (27.3%), treatment-naive (62.5%) and HIV negative injection heroin users (43.5%) had AST to ALT ratio ≥2.0, indicating increased liver damage in both HIV infected and uninfected injection heroin users.

Albumin to total protein ratio differed in the groups (*P* < 0.0001) and were significantly lower in the anti-retroviral treatment-exposed (median, 0.5; IQR, 0.1) and treatment-naive (median, 0.4; IQR, 0.2) groups compared to HIV negative injection heroin users (median, 0.6; IQR, 0.1) and healthy controls (median, 0.6; IQR, 0.1; *P* < 0.01 and *P* < 0.0001, respectively). Likewise, albumin to globulin ratio varied in the study groups (*P* < 0.0001) such that the anti-retroviral treatment-experienced (median, 1.0; IQR, 0.5) and naive (median, 0.8; IQR, 0.6) individuals had lower values in comparison to HIV negative group (median, 1.5; IQR, 0.4; *P* = 0.002) and healthy controls (median, 1.4; IQR, 0.3; *P* < 0.0001).

APRI differed significantly across the study groups (*P* = 0.005) with the anti-retroviral treatment-exposed group (median, 0.25; IQR, 0.20) having higher values compared to the healthy controls (median, 0.14; IQR, 0.10; *P* = 0.001). Moreover, the frequency of moderate-to-severe forms (i.e., APRI ≥ 0.5) of liver damage was only noted in anti-retroviral treatment-exposed (18.2%) and -naive (6.3%) individuals (Table 2), suggesting increased liver derangements in HIV infected injection heroin users.

### Correlation of the clinical chemistry markers with CD4+ T cells and HIV viral loads

**Figure 1:**
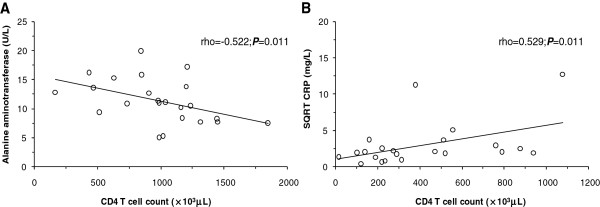
**Correlations of CD4+ T cell counts with alanine aminotransferase and C-reactive protein.** Correlations of the CD4+ T cells with alanine aminotransferase and C-reactive protein levels were determined using the Spearman’s rank correlation test. **(A)** Correlation between CD4+ T cells and alanine aminotransferase in HIV uninfected injection heroin users (n = 23). **(B)** Correlation between CD4+ T cells and square root of the C-reactive protein (SQRT CRP) in HIV infected ART-exposed injection heroin users (n = 22).

CD4+ T cells inversely correlated with ALT levels (ρ = −0.522; *P* = 0.011; Figure 1A) in HIV uninfected injection heroin users and positively with the CRP levels (ρ = 0.529; *P* = 0.011; Figure 1B) in HIV infected anti-retroviral treatment-experienced injection heroin users.

**Figure 2:**
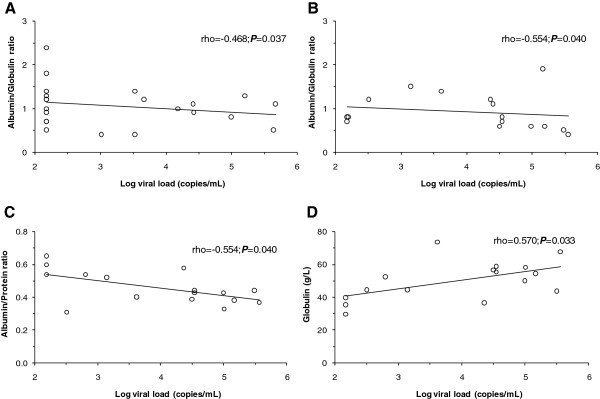
**Correlations of HIV-1 viral load with albumin to globulin, albumin to total protein and globulin.** Correlations of the HIV-1 viral loads with albumin to globulin, albumin to total protein and globulin levels were determined using the Spearman’s rank correlation test. **(A)** Correlation between HIV-1 viral load and albumin to globulin index in anti-retroviral treatment-exposed injection heroin users (n = 22). **(B)** Correlation between HIV-1 viral load and albumin to globulin index in anti-retroviral treatment-naive injection heroin users (n = 16). **(C)** Correlation between HIV-1 viral load and albumin to total protein index in anti-retroviral treatment-naive injection heroin users (n = 16). **(D)** Correlation between HIV-1 viral load and calculated globulin in anti-retroviral treatment-naive injection heroin users (n = 16).

HIV-1 viral loads inversely correlated with albumin to globulin (ρ = −0.468, *P* = 0.037; Figure 2A) ratio in HIV infected anti-retroviral treatment-exposed injection heroin users. In addition, the HIV-1 viral loads inversely correlated with albumin to globulin (ρ = −0.554; *P* = 0.040; Figure 2B) and the albumin to total protein (ρ = −0.554; *P* = 0.040; Figure 2C) values, and positively with globulin (ρ = 0.570; *P* = 0.033; Figure 2D) in HIV infected anti-retroviral treatment-naive injection heroin users.

## Discussion

Clinical chemistry laboratory analysis constitutes a key step in patient assessment for initiating and monitoring response to anti-retroviral treatment [5]. The reliability and accuracy of clinical chemistry analytes, however, is confounded by complex interaction between injection drug use and anti-retroviral drugs. Therefore, this cross-sectional clinical laboratory study determined the clinical chemistry markers in HIV infected and uninfected injection heroin users from coastal Kenya.

Absolute albumin levels and albumin to total protein ratio reductions observed in the HIV-1 infected injection drug users are indications of derangements in protein metabolism. The decreases in the albumin levels can be attributed to low dietary intake of proteins that is linked to low synthesis of albumin [19], a feature of malnutrition that is frequently observed among injection drug users presenting with and without HIV infection [20]. It is also possible that suppression of albumin results from inhibition of hepatic synthesis at abusive doses of heroin [15]. In addition, opioid-induced hepatotoxicities and inflammatory-mediated hepatic damage cause reduced hepatic synthetic functions [15,21]. Thus, findings presented here suggest that injection heroin use among HIV-infected individuals promote increased reductions in the albumin levels and marked alterations in the albumin to total protein ratio.

The inverse associations of HIV viral load and albumin to total protein and albumin to globulin indices in the anti-retroviral-naive injection heroin users signify disease progression but during treatment the albumin to globulin ratio is inverted indicating reductions in the viral loads and immune reconstitution. This premise is, in part, consistent with clinical studies in HIV infected patients commencing highly active anti-retroviral therapy showing that hypoalbuminaemia is associated with morbidity and mortality [22,23]. Therefore, reduced albumin and low albumin to total protein ratio are important laboratory measures in injection heroin users that can be utilized as important surrogates for screening and for initiating and monitoring of anti-retroviral treatment in HIV infected injection heroin users.

In contrast to previous studies, a higher globulin level was found in the HIV infected injection heroin users naive for anti-retroviral treatment [17]. This finding suggests that hyperglobulinaemia is an important laboratory marker of HIV infected patients injecting illicit drugs. Hyperglobulinaemia characterizes B cell dysfunction during primary HIV infections and persisting into chronic infections [24]. The lower median albumin to globulin values in the presence of higher proportions of ratios ≤1.0, and the correlations of HIV viral loads with globulin in the anti-retroviral treatment-naive individuals, further corroborate increased derangements in B cell functions in both HIV infected and uninfected injection heroin users. The findings of the current study also parallel studies showing elevated globulin and the albumin to globulin ratio in Australian drug addicts [12]. In addition, elevations in total IgM and IgG, specific anti-morphine IgM, and cross-reactive IgM auto-antibodies were recorded in HIV infected and uninfected opioid users [25-27]. Hence, it appears heroin-induced antibodies promote inflammation leading to liver damage in injection users. Elevated globulin with concomitant lower albumin to globulin ratio may thus be a useful biomarker for clinical laboratory assessment of HIV infected and uninfected injection heroin users.

Reduced absolute ALT levels found in the HIV sero-negative injection heroin users, indicate reduced hepatic functionality. However, the higher AST to ALT ratio and proportions of AST to ALT ratio ≥2.0 in the HIV sero-negative and infected injection heroin users naive or on anti-retroviral treatment, suggest synergistic heroin and anti-retroviral drug-induced hepatic inflammation. These results are similar to observations showing that the AST to ALT ratio is a better measure of liver enzyme activity [28]. In addition, the results are, in part, supported by the elevated AST, AST to ALT ratios, and higher proportions of ALT observed in patients on anti-retroviral treatment [10-12]. Since a large number of the injection heroin users in the present study were concomitantly consuming alcohol and *khat*, it is possible that these non-injection substances synergistically promote the alterations in the ALT levels [10,29]. Importantly, the inverse correlations of absolute ALT levels and the CD4+ T cell counts in the HIV sero-negative injection heroin users, suggests that the CD4+ T cell count can also be utilized as a surrogate marker of liver function in the management of HIV negative heroin users.

The greater magnitude APRI median values and proportions ≥0.5 in the HIV infected injection heroin users on anti-retroviral treatment, suggests, that APRI is a better screening indicator of heroin and anti-retroviral treatment in this population. This observation is, in part, parallel to the time-dependent increases and prognostic value of APRI among HIV and hepatitis C co-infected patients undergoing anti-retroviral treatment [7], and studies in injection drug users showing the utility of APRI in predicting hepatic fibrosis [30]. It is, therefore, likely that increased hepatotoxicity in HIV patients on anti-retroviral treatment results from the synergistic effect of anti-retroviral drugs, heroin and poly-substance consumption. Hence, APRI and proportions of APRI ≥0.5 are important measures for evaluating the degree of hepatoxicity in HIV infected injection heroin users undergoing anti-retroviral treatment.

Elevations of the C-reactive protein in all the study groups of injection heroin users is similar to previous studies showing elevated C-reactive protein in Australian drug addicts [12], and higher C-reactive protein levels in buprenorphine injection users in Singapore [31]. Higher levels and proportions of individuals of this acute phase protein in both HIV infected and uninfected injection heroin users in the present study are indicative of increased liver synthesis following consumption of the illicit drugs, infection and cytokine release [32]. In addition, the increases indicate heightened liver damage through illicit drug- and anti-retroviral treatment-mediated necrosis, apoptosis, and immune mechanisms. Of significance are analyses showing positive correlations between the C-reactive protein levels and the CD4+ T cell count in injection heroin users on anti-retroviral treatment. This result may reflect development of immune reconstitution inflammatory syndrome (IRIS) in the injection heroin users on anti-retroviral treatment resulting from poor adherence and illicit drug use. Consistent with this hypothesis, previous studies illustrated a link between IRIS and low adherence to anti-retroviral therapy, alcohol use, and low suppression of viral load among South African adults initiating anti-retroviral therapy [33]. Implications of this observation include utilization of the C-reactive protein in assessing immune reconstitution following initiation of anti-retroviral treatment in injection heroin users.

Our results clearly show high rates of poly-drug use among injection heroin users in Mombasa. It is highly likely that the complex interactions of opioids and/or active compounds in bhang (Δ9-tetrahydrocannabinol), cigarettes (nicotine), rohypnol (benzodiazepines), alcohol, khat (cathinone), and anti-retroviral drugs promote the occurrence of drug dependence and adverse events. With regards to clinical chemistry profiles, such complex interactions induce hepatic metabolic derangements leading to toxicity and altered profiles of clinical chemistry markers. Consistent with these propositions, previous studies among drug users showed that interactions between opioids and benzodiazepines or alcohol increase occurrence of adverse events, overdose and death [34], as nicotine increase rates of opioid consumption [35]. In addition, drugs of abuse (alcohol, opioids, benzodiazepines, marijuana, and nicotine) reduce the efficacy of anti-retroviral drugs leading to toxicity, treatment failure and high viral loads [36]. Taken together, poly-drug use appears to promote toxicity leading to altered clinical chemistry profiles in both HIV-1 infected and uninfected injection drug users.

While a prospective design would have been important in examining the utility of clinical chemistry markers, in response to drug rehabilitation and HIV treatment, this cross-sectional study provides the first baseline information for screening of injection drug users for initiating and monitoring anti-retroviral treatments in Kenya. Although recruitment into this study was based on self-reported injection heroin use, it is possible that the study participants were also using other opioids, hence the need to carry out toxicological analyses to provide additional insights into the complex interactions between injection drugs and anti-retroviral treatment. These collectively can be linked to clinical laboratory markers and patient prognosis in a prospective approach.

## Conclusions

This study provides further evidence that chronic inflammation in HIV infected and uninfected injection heroin users is characterized by derangements in hepatic synthetic functions. Our data suggest that injection heroin, HIV infection and anti-retroviral treatment differentially alter ALT; albumin; APRI and AST to ALT, albumin to total protein and albumin to globulin indices; C-reactive protein; and globulin in injection drug users.

## Competing interests

None of the authors have a commercial relationship or financial conflict of interest as part of this study.

## Authors’ contributions

TW and VB conceived and designed the study, and performed the experiments, and along with AA, JW, CO, TMK, FO, and JNK designed the study. TW performed statistical analyses and interpretation of data, and co-drafted the manuscript with EM and VB. EPM and CO critically revised the manuscript. All authors have read and approved the manuscript.

## Pre-publication history

The pre-publication history for this paper can be accessed here:

http://www.biomedcentral.com/1472-6890/14/32/prepub
